# Harnessing *Trichoderma* Species for Sustainable Biocontrol: Mechanisms, Formulation Strategies, Commercialization, and Field Applications

**DOI:** 10.3390/plants15152260

**Published:** 2026-07-23

**Authors:** Sidratul Muntaha Binta Anam Otithi, Md. Sohel Rana, Md. Shariful Islam, Randa Mohammed Zaki, Sajad Ali, Muhammad Fazle Rabbee, Md. Mohidul Hasan, Kwang-Hyun Baek

**Affiliations:** 1 Department of Plant Pathology, Hajee Mohammad Danesh Science and Technology University, Dinajpur 5200, Bangladesh; 2 Department of Genetics and Plant Breeding, Bangladesh Agricultural University, Mymensingh 2202, Bangladesh; 3 Department of Pharmaceutics, College of Pharmacy, Prince Sattam Bin Abdulaziz University, Al Kharj 11942, Saudi Arabia; 4 Department of Pharmaceutics and Industrial Pharmacy, Faculty of Pharmacy, Beni-Suef University, Beni-Suef 62511, Egypt; 5 Department of Biological Sciences, College of Science, King Faisal University, Al-Ahsa 31982, Saudi Arabia; 6 Department of Biotechnology, Yeungnam University, Gyeongsan 38541, Gyeongsangbuk-do, Republic of Korea

**Keywords:** *Trichoderma* spp., biocontrol agents, field application strategies, sustainable agriculture

## Abstract

*Trichoderma* species are widely investigated and commercially applied as eco-friendly biocontrol agents in sustainable agriculture. These filamentous fungi protect plants through multiple complementary mechanisms, including mycoparasitism, antibiosis, competition for nutrients and ecological niches, and induction of systemic resistance in host plants. These activities are mediated by a diverse array of secondary metabolites, hydrolytic enzymes, and signaling pathways that collectively suppress pathogens and enhance plant health. Beyond disease control, selected *Trichoderma* strains promote plant growth by improving nutrient acquisition, modulating phytohormone signaling, and increasing tolerance to abiotic stresses. This review summarizes recent advances in the mechanisms underlying *Trichoderma* spp. mediated biocontrol, with particular emphasis on secondary metabolites, formulation strategies, commercialization, and field applications. Commercial products are available in various formulations, including wettable powders, granules, and liquid preparations, and have demonstrated efficacy against several economically important plant diseases under field conditions. However, their performance remains highly dependent on strain characteristics, host species, environmental conditions and agricultural practices, resulting in inconsistent efficacy across agroecosystems. Recent progress in genomics, transcriptomics, and metabolomics has substantially improved our understanding of *Trichoderma*–plant–pathogen interactions and revealed considerable strain-specific variation in biocontrol and plant growth-promoting traits. Future research should prioritize strain-specific optimization, formulation stability, microbiome-informed applications, and improved field predictability. Overall, *Trichoderma* spp. Represents a valuable component of integrated disease management, offering an effective and sustainable alternative to synthetic pesticides.

## 1. Introduction

The Food and Agriculture Organization (FAO) estimates that global food production must increase by ~70% by 2050 to meet the demands of a growing population. At the current growth rate, the global population is projected to reach ~9.1 billion by 2050 [1,2]. However, recurrent plant disease outbreaks pose a significant threat to global food security and environmental sustainability, a challenge further intensified by climate change [3]. To mitigate yield losses and achieve short-term production targets, many farmers increasingly depend on synthetic fertilizers and chemical fungicides. A global assessment reveals that 88 countries collectively consumed ~110 million tons of NPK fertilizers annually between 1961 and 2010, with China identified as the largest consumer. Insecticide consumption was highest in the United States, followed by India and Brazil, while pesticide use increased significantly in countries such as Bangladesh, Thailand, Ghana, Ethiopia, and Burkina Faso [4]. While these agrochemical inputs have significantly increased crop productivity, their prolonged and excessive use has raised significant environmental and human health concerns [5]. Synthetic fertilizers and pesticides can disrupt beneficial soil microbiota, including nitrogen-fixing rhizobacteria and phosphorus-solubilizing bacteria, thereby compromising nutrient cycling and soil fertility [4]. The Green Revolution of the 20th century, characterized by the adoption of high-yielding crop varieties, chemical fertilizers, pesticides, and disease-resistant cultivars, significantly enhanced agricultural productivity. However, it also produced unintended consequences, including biodiversity loss, increased dependence on water and fossil fuels, and widespread environmental degradation associated with excessive nitrogen inputs [6]. In this context, biological control agents represent a sustainable alternative by reducing dependence on synthetic agrochemicals, minimizing ecological damage, and offering long-term disease management strategies.

*Trichoderma* spp. are among the most widely studied and effective fungal biocontrol agents, owing to their opportunistic behavior, cosmopolitan distribution, and multifunctional roles in agroecosystems. Beyond suppressing phytopathogens and pests, they also serve as important model organisms for advancing research in agricultural and environmental sciences [7]. These ubiquitous fungi occur widely in soil, air, and plant-associated environments and exhibit multiple plant-beneficial traits. *Trichoderma* spp. suppress phytopathogens, enhance nutrient uptake, promote plant growth, strengthen plant immune responses, and assist in remediating agrochemical-contaminated soils [8,9]. Diverse *Trichoderma* spp. have been identified as endophytes in numerous plant species, colonizing various plant tissues and promoting plant health through multiple mechanisms, including antifungal activity, growth promotion, and enhanced tolerance to biotic and abiotic stresses. Notable species include *T. aggressivum*, *T. alni*, *T. effusum*, *T. hamatum*, *T. koningiopsis*, and *T. nivale* [10]. For example, *Trichoderma atroviride*—an endophytic fungus isolated from the rhizosphere of *Salvia miltiorrhiza*—stimulates hairy root formation and enhances the biosynthesis of valuable secondary metabolites, including tanshinones [11].

**Figure 1:**
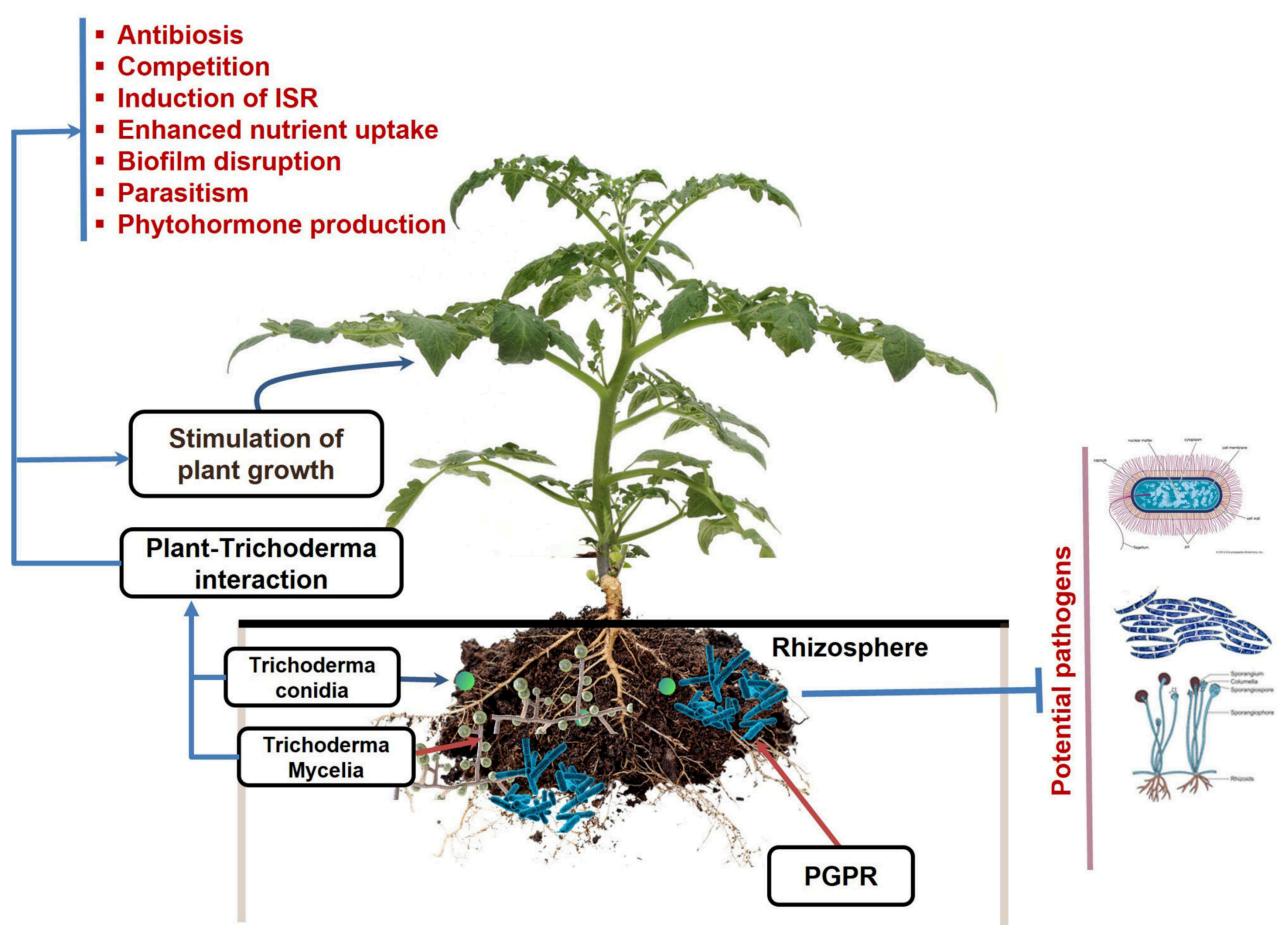
Schematic representation of the biocontrol and plant growth-promoting mechanisms of *Trichoderma* spp. in the rhizosphere. Colonization of plant roots by *Trichoderma* spp. via conidia and mycelia enhances plant development by improving nutrient uptake, phytohormone production, and ISR. Biocontrol mechanisms include antibiosis, competition for nutrients and space, mycoparasitism, biofilm disruption, and suppression of soil-borne pathogens. Interactions with PGPR further support plant health and growth. Abbreviations: PGPR, plant-growth-promoting rhizobacteria; ISR, induced systemic resistance.

*Trichoderma* spp., belonging to the family Hypocreaceae, are among the most common culturable fungal genera in soil ecosystems. Members of this genus are soil-dwelling, free-living, filamentous fungi that reproduce primarily asexually and tolerate facultative anaerobic conditions [12,13]. Beyond disease suppression, these fungi promote plant growth by modifying the rhizosphere, competing effectively for nutrients, thriving under adverse soil conditions, and activating plant defense pathways (Figure 1) [2,12,14]. Moreover, *Trichoderma* spp.) is cosmopolitan and can inhabit semi-aquatic ecosystems, including mangroves, where they maintain high population densities [15,16]. For over seven decades, *Trichoderma* spp. have been recognized for producing antibiotics and various secondary metabolites with potent antagonistic activity against pathogenic organisms [12].

Recent studies significantly advance our understanding of *Trichoderma*–plant interactions across various plant-associated niches, including the rhizosphere, endosphere, and phyllosphere, with these fungi exhibiting distinct colonization strategies and functional roles. However, phyllospheric *Trichoderma* spp. remain considerably less studied than their rhizospheric and endophytic counterparts [17]. They exhibit strong rhizocompetence and utilize species-specific biocontrol mechanisms, including mycoparasitism, production of cell wall–degrading enzymes, competition for nutrients and space, and synthesis of fungistatic and plant-growth-promoting secondary metabolites [12]. These mechanisms further contribute to induced systemic resistance, enhanced antioxidant activity, improved photosynthetic efficiency, increased nutrient uptake, and nutrient solubilization [18]. Among the most extensively investigated *Trichoderma* spp. are *T. viride*, *T. asperellum*, *T. atroviride*, *T. harzianum*, *T. longibrachiatum*, and *T. virens*. Of these, the first three are widely used as commercial biocontrol agents owing to their dual modes of action, involving mycoparasitism and competitive exclusion of phytopathogenic fungi (Table 1) [19]. Notably, *Trichoderma* spp. constitute the active biological agents in approximately 60% of commercially available biofungicides, highlighting their significant role in the development of sustainable disease management strategies [10].

## 2. *Trichoderma* spp. as Broad-Spectrum Biocontrol Agents

Fungal phytopathogens cause >40% of plant diseases globally, representing a major constraint to agricultural productivity [31]. *Trichoderma* spp. are well known for their ability to inhibit the growth and proliferation of various phytopathogenic fungi, including *Alternaria* spp., *Acremonium cucurbitacearum*, *Aphanomyces cochlioides*, *Aspergillus* spp., *Botrytis cinerea*, *Fusarium oxysporum*, *Lasiodiplodia theobroma*, *Phoma betae*, *Pythium* spp., *Rhizoctonia solani*, *Rhizopus oryzae*, *Sclerotium rolfsii*, and *Verticillium dahliae* [19,32]. Owing to this broad antagonistic potential, *Trichoderma* spp. have been successfully applied as biocontrol agents across diverse crops, including citrus, maize, cucumber, potato, rice, strawberry, grapevine, soybean, tomato, and wheat [33,34]. For example, *T. harzianum* is effectively used to control foliar pathogens of cucumber, including *B. cinerea*, *Pseudoperonospora cubensis*, *Sclerotinia sclerotiorum*, and *Sphaerotheca fusca* [35]. Similarly, *T. asperellum* demonstrates strong suppression of soilborne pathogens, particularly *F. oxysporum*, in potato and tomato cropping systems [36]. Wheat powdery mildew (WPM), caused by the fungal pathogen *Blumeria graminis* f. sp. *tritici*, is one of the most devastating diseases threatening global wheat production. The efficacy of *Trichoderma* spp. in controlling WPM at the seedling stage was evaluated across 35 genetically diverse wheat genotypes. Significant differences in disease resistance are observed among the four treatment groups, confirming the ability of *Trichoderma* spp. to suppress WPM. Among the tested species, *T. asperellum* T34 is the most effective, reducing disease severity by 50.56% [37].

## 3. Mechanisms Underlying the Beneficial Effects of *Trichoderma* spp.

The principal mechanisms underlying *Trichoderma* spp. biocontrol activity include antibiosis, mycoparasitism, induction of systemic or localized plant resistance, and competition for space and nutrients [38]. These antagonistic fungi, commonly isolated from soil environments, are widely utilized as biofertilizers, biopesticides, and soil amendments owing to their ability to produce various biologically active metabolites and cell wall–degrading enzymes (Table 2). *Trichoderma* spp. secrete diverse effectors, including proteins, small RNAs, and secondary metabolites, particularly volatile organic compounds (VOCs), play key roles in regulating plant growth, immunity, and stress responses during plant–microbe interactions [39]. *Trichoderma* spp. antagonize plant pathogens through both direct and indirect mechanisms, collectively referred to as their biocontrol capacity. Direct biocontrol mechanisms include antibiosis and mycoparasitism, whereas indirect mechanisms include induction of plant systemic resistance, competition for nutrients and niches, and plant growth promotion, thereby reducing disease incidence and severity [40]. Root colonization by *Trichoderma* spp. further enhances root architecture, improves nutrient uptake, increases crop productivity, and strengthens plant tolerance to biotic and abiotic stresses [40]. Advances in genomic and proteomic analyses reveal numerous genes and proteins involved in the complex tripartite interactions among plants, *Trichoderma*, and phytopathogens, providing valuable insights into their multifunctional behavior [41]. A deeper understanding of these mechanisms—including antibiosis, mycoparasitism, activation of plant defense pathways, nutrient competition, and efficient rhizosphere and root colonization—is essential for developing superior biocontrol strains and ensuring their effective application in sustainable agriculture [42].

### 3.1. Diversity of Secondary Metabolites Produced by Trichoderma spp.

**Figure 2:**
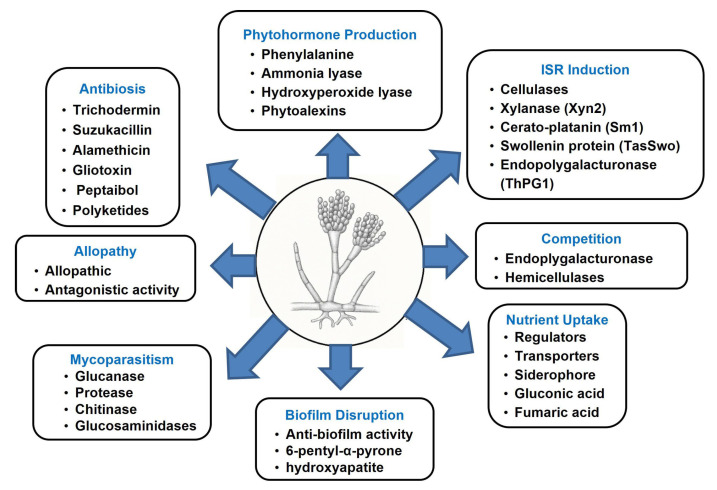
*Trichoderma* spp.-based secondary metabolites contribute to biocontrol activity and plant growth promotion.

Secondary metabolites produced by *Trichoderma* spp. exhibit remarkable chemical diversity. Terpenoids constitute the dominant class of reported *Trichoderma* secondary metabolites (approximately 52%), followed by polyketides (approximately 27%), whereas peptides (particularly peptaibols), alkaloids, steroids, and other metabolite classes are less abundant [46]. Collectively, these compounds exhibit a broad spectrum of biological activities, including antibacterial and antifungal effects. More than 370 *Trichoderma* spp. have been identified to date. Among them, several species were extensively studied and widely recognized for their potential as biological control agents, including *T. asperellum*, *T. hamatum*, *T. viride*, *T. koningii*, *T. polysporum*, and *T. harzianum* [8]. Their sources include algae, endophytic niches, marine sponges, soil, sediments, nests, mushrooms, sea stars, soft corals, and other marine environments (32.98%, 17.16%, 10.99%, 10.72%, 6.17%, 5.90%, 4.83%, 2.68%, 1.88%, and 2.41%, respectively), such as seawater, marine tunicates, and fish gastrointestinal tracts [47,48]. *Trichoderma* spp. biosynthesize a structurally diverse array of polyketide secondary metabolites, including trichoderones A-B, koninginin A, Cytosporones Y and Z (Figure 2) [46,49]. These compounds exhibit a broad spectrum of biological activities, including antifungal, antimicrobial, phytostimulatory, and plant defense-inducing effects, thereby contributing to biocontrol efficacy and the plant-associated lifestyle of *Trichoderma*. The majority of these metabolites are assembled by iterative Type I polyketide synthases, which are encoded within dedicated polyketide biosynthetic gene clusters (BGCs) [50,51,52]. Expression of these BGCs is often silent under standard laboratory conditions but can be activated in response to environmental stimuli, including stress or in situ interactions with phytopathogens, leading to induced metabolite production during co-cultivation or competitive challenge [25].

The velvet complex of *Trichoderma* spp. is a conserved multi-protein regulatory system, typically forming a heterotrimeric complex, that serves as a global regulatory switch controlling fungal development and metabolism. It integrates environmental and developmental signals to regulate secondary metabolite biosynthesis, coordinate the asexual-to-sexual reproductive transition, and modulate key traits associated with biocontrol efficacy. In *Trichoderma* spp., velvet complex components play a pivotal role in mycoparasitism, antagonistic interactions with phytopathogens, and regulating hydrolytic enzyme production, including cellulases, which are essential for ecological competitiveness and industrial applications [53,54]. Gliotoxin—produced by *T. virens* GL 20—was the first biocontrol agent commercially developed and marketed as SoilGard^®^, providing effective protection against root and crown rot diseases caused by soilborne pathogens, such as *Pythium* and *Rhizoctonia* spp. [55]. Volatile and non-volatile metabolites produced by *Trichoderma* spp. are capable of inhibiting fungal mycelial growth. Antibiotics, such as trichodermin, trichodermamide, suzukacillin, and alamethicin, primarily synthesized by *Trichoderma* spp., enhance membrane permeability by disrupting pathogen cell membranes, thereby contributing to their antifungal efficacy [56]. *T. viride* and *T. harzianum* have been reported to significantly suppress radial mycelial growth of several pathogenic fungi. Several other species, including *T. aureoviride*, *T. harzianum*, *T. koningii*, *T. virens*, *T. hamatum*, and *T. lignorum*, are known to produce various antibiotics, bioactive metabolites, and fungicidal compounds. Producing these metabolites is tightly regulated by BGCs and associated transcription factors, leading to the synthesis of chemically diverse classes, including anthraquinones, simple pyrones, peptaibols, polyketides, sesquiterpenes, and alkaloids. Collectively, *Trichoderma*-derived metabolites exhibit potent antibacterial, antifungal, and insecticidal activities, highlighting their ecological significance and biotechnological potential (Table 3) [57].

### 3.2. Secondary Metabolites of Trichoderma spp. in Biofilm Degradation

Biofilms are structured, surface-attached communities of microorganisms encased in a self-produced extracellular polymeric substance (EPS) matrix. This slime-like matrix acts as an activated matrix that allows microbes to share nutrients and physically defend themselves against environmental stresses, such as antibiotics, desiccation, and detergents [65]. Polysaccharides, proteins, nucleic acids, and lipids are essential biopolymers for microbial adherence and survival [66]. EPS attaches microorganisms to surfaces, maintaining cellular integrity. *Trichoderma* produces enzymes that disrupt the biofilm of other pathogenic microorganisms. This process, known as anti-biofilm activity, is a notable feature of *Trichoderma*. The secondary metabolite 6-pentyl-α-pyrone, produced by *T. atroviride* P1 in combination with mineral hydroxyapatite, is used to evaluate the lytic activity of the Xccφ1 phage, specifically targeting *Xanthomonas campestris* pv. *campestris* [67]. Under in vitro conditions, 7-day-old cultures of *T. asperellum* were reported to inhibit biofilm formation by *Burkholderia pseudomallei*—a soil- and water-dwelling bacterium that causes human disease—during the adhesion phase and early biofilm development. Treatment with *T. asperellum* resulted in approximately six-fold and three-fold reductions in biofilm formation, respectively, at a concentration of 1 × MIC (~7.81 mg/mL). However, the extent to which these inhibitory effects occur in soil or plant-associated environments remains uncertain because microbial interactions and environmental conditions can substantially influence biofilm formation and inhibition [68]. Furthermore, another study demonstrates that biofilm-based formulations combining *Trichoderma* and *Azotobacter* are more effective than single microbial inoculants in stimulating plant defense responses in wheat and cotton seedlings. Application of these biofilms markedly enhances the activities of key defense-related enzymes, including peroxidase, polyphenol oxidase, catalase, and PAL, along with increased total phenolic content. These findings suggest that the synergistic interaction among microorganisms within a biofilm matrix efficiently activates ISR, thereby strengthening antioxidant capacity and overall plant defense against environmental stresses [69].

### 3.3. Mycoparasitism by Trichoderma spp.

*Trichoderma* spp. parasitize a range of other fungal pathogens through mycoparasitism [12], a process involving a sequence of coordinated events that enable the fungus to attack its target. Initially, the mycoparasite grows toward the target fungus and secretes chitinolytic enzymes, which weaken and degrade the cell wall of the pathogen. Upon contact, *Trichoderma* spp. adhere to and coil around the host hyphae, followed by enzymatic degradation and penetration. This mycoparasitic activity suppresses pathogen growth, thereby preventing the development of plant diseases [17,70]. *Trichoderma* spp. are highly effective in reducing pathogen inoculum levels, particularly for soil-borne diseases, where they are commonly applied. Manzar et al. (2022) report that mycoparasitic strains exhibit significantly higher disease suppression than the mechanisms based solely on induced defense responses or antibiosis [38]. Many *Trichoderma* spp. exhibit mycoparasitic activity against a broad range of phytopathogenic fungi, including *Pythium*, *Phytophthora*, *Rhizoctonia*, and *Peronospora* spp. [12,71]. During these interactions, pathogen hyphae frequently undergo morphological alterations, including swelling, shortening, deformation, cytoplasmic shrinkage, and cell-wall rupture. These changes result from hyphal attachment, coiling, penetration, and the secretion of cell wall-degrading enzymes and antifungal metabolites, ultimately leading to pathogen death [8,72]. This mycoparasitic activity is mediated by the secretion of cell wall-degrading enzymes, including chitinases, β-1,3 glucanases, cellulases, and proteases, efficiently degrading the structural components of host pathogens [70,71]. Experimental studies clearly demonstrate this behavior in vitro, with *T. koningiopsis* and *T. virens* actively attacking and degrading the hyphae of *Colletotrichum siamense*, the causal agent of anthracnose in pecan [70].

At the molecular level, several *Trichoderma* spp. exhibit differential gene expressions encoding proteases and oligopeptide transporters before and during contact with *R. solani*. Expressed sequence tag (EST) analyses have revealed the early induction of subtilisin-like serine proteases at the onset of *Trichoderma*–pathogen interactions. The *prb1* gene, which encodes an extracellular protease, plays a crucial role in enhancing the mycoparasitic activity of *Trichoderma* spp. against *Botrytis cinerea* [73,74]. Furthermore, certain G-protein-coupled receptor–encoding genes in *Trichoderma* spp. mediate pathogen recognition and activation of mycoparasitic signaling cascades, including G-proteins, adenylate cyclase, and mitogen-activated protein kinase (MAPK) pathways. These signaling networks regulate the expression of hydrolytic enzymes, secondary metabolites, and other factors that contribute to pathogen cell-wall degradation and lysis. Among these signaling components, the MAP kinase Tmk1 in *T. atroviride* plays a central role in regulating mycoparasitism by controlling the expression of genes associated with signal transduction, cell wall integrity, siderophore biosynthesis, oxidoreductase activity, protein folding, and detoxification [75].

### 3.4. Induction of Systemic Resistance in Plants by Trichoderma spp.

Plants activate systemic acquired resistance (SAR) following localized pathogen infection to enhance resistance in distal, uninfected tissues and limit the systemic spread of pathogens. SAR is typically initiated after the hypersensitive response (HR), which involves localized cell death at the site of primary infection. This systemic defense response is characterized by the accumulation of salicylic acid (SA), activation of SA-dependent signaling pathways, induction of pathogenesis-related (PR) genes, and accumulation of PR proteins [76]. Induced systemic resistance (ISR) is triggered by pathogenic attacks and root colonization and interactions with beneficial microbes, including systemic mutualists and plant growth-promoting rhizobacteria [77].

*Trichoderma* spp.-induced root colonization triggers ISR through the recognition of fungal microbe-associated molecular patterns (MAMPs) by plant pattern recognition receptors (PRRs). These MAMPs include chitin oligomers, β-glucans, and secreted fungal elicitor proteins, while colonization may also stimulate the release of plant-derived damage-associated molecular patterns (DAMPs), further enhancing immune signaling [41]. MAMPs are conserved microbial molecules recognized by pattern-recognition receptors located on the plasma membrane, representing the first step of ISR activation by beneficial microbes, such as *Trichoderma* spp. DAMPs are endogenous plant-derived molecules released upon cell damage, wounding, or cell wall modification. In contrast to MAMPs, DAMPs are produced by the host plant. Their recognition triggers rapid defense responses, including reactive oxygen species (ROS) production, Ca^2+^ influx, MAPK activation, and callose deposition. These early signaling events subsequently activate jasmonic acid (JA), ethylene (ET), and salicylic acid (SA) signaling pathways, leading to the induction of defense-related genes, such as *PR3*, *PR4*, *PDF1.2*, *PAL*, and *PAD4*, with NPR1 serving as a key regulator of systemic defense signaling [78].

**Figure 3:**
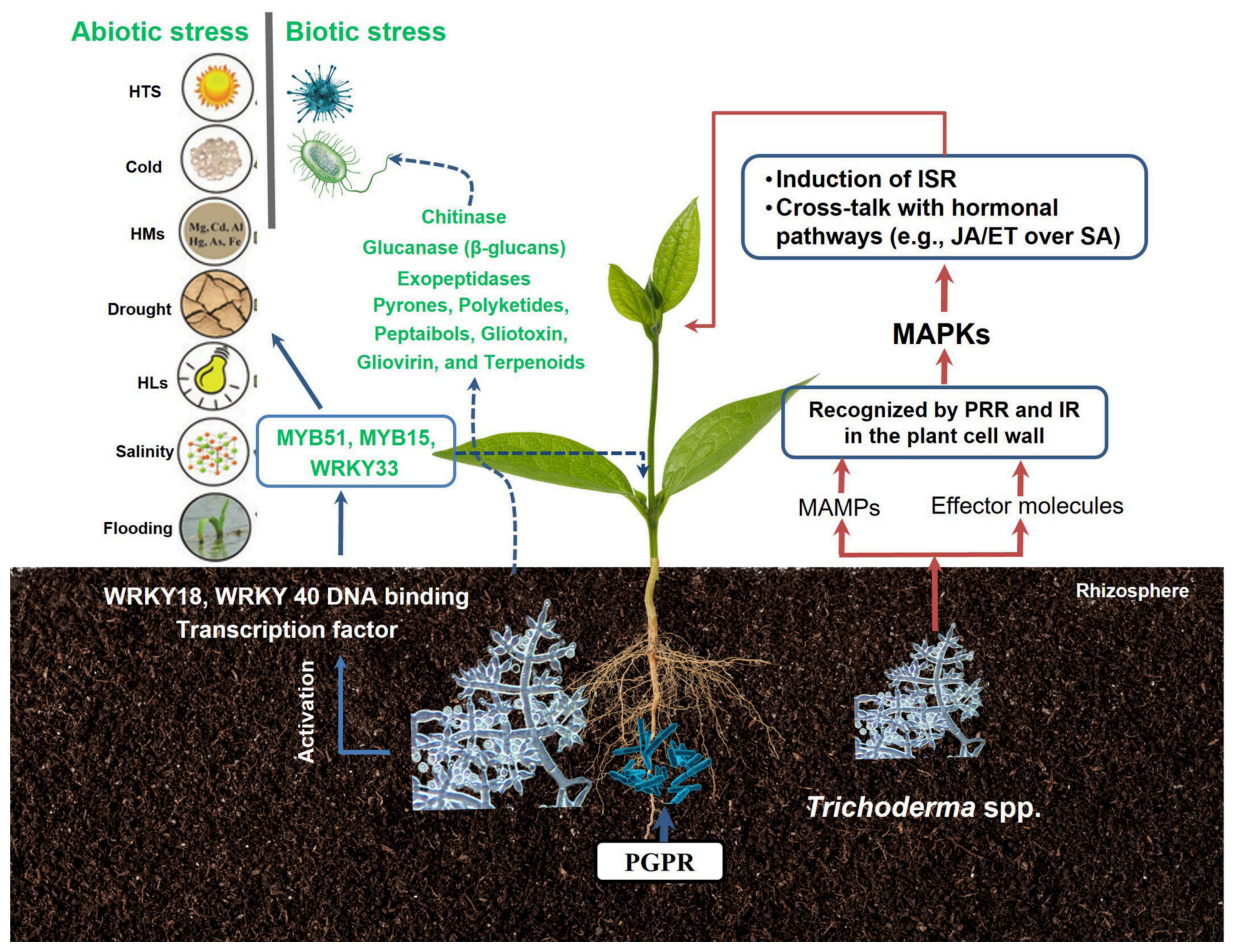
Mechanisms by which *Trichoderma* spp. enhance plant tolerance to abiotic and biotic stresses through rhizosphere colonization and immune signaling. *Trichoderma* spp. colonize the rhizosphere and produce diverse bioactive molecules, such as polyketides, terpenoids, pyrones, peptaibols, gliotoxin, gliovirin, and enzymes, such as chitinases, β-glucanases, and exopeptidases. These microbial-derived molecules are recognized by PRRs and IRs as MAMPs and trigger MAPK signaling cascades and induce ISR. Crosstalk among phytohormonal signaling pathways, particularly JA and ET, together with SA, regulates the activation of defense-related transcription factors, including WRKY18, WRKY40, MYB33, MYB15, and WRKY51. These signaling networks enhance plant tolerance to abiotic stresses (heat, cold, heavy metals, drought, high light, salinity, and flooding) and biotic stresses, while strengthening resistance against a broad range of plant pathogens. Abbreviations: ET, ethylene; HLs, high light stresses; HMs, heavy metals; HTS, heat stress; IRs, immune receptors; ISR, induced systemic resistance; JA, jasmonic acid; MAPK, mitogen-activated protein kinase; MAMPs, microbe-associated molecular patterns; PGPR, plant growth-promoting rhizobacteria; PRRs, pattern recognition receptors; SA, salicylic acid.

Concurrently, pathogen challenge activates the SA signaling pathway, resulting in nuclear accumulation and activation of NPR1 and the subsequent induction of PR genes, including *PR1*, *PR2*, *PR5*, and *PAL*, hallmarks of SAR. NPR1 functions as a central regulatory hub integrating SA- and JA/ET-dependent signaling by suppressing JA responses in the cytosol while promoting SA-mediated defense gene expression in the nucleus. Root association with *Trichoderma* induces systemic and local defense responses that limit further colonization by other harmful microorganisms. While colonization is often restricted to the roots, these systemic signals protect plants from foliar pathogens [12]. Plant recognition of *Trichoderma* involves detecting MAMPs, which are present in *Trichoderma* proteins released during cell colonization, including cellulases, xylanase Xyn2, cerato-platanin Sm1, swollenin protein TasSwo, and endopolygalacturonase ThPG1. Interaction with these MAMPs, such as elicitor protein 1 (Epl1) from *Trichoderma* spp. trigger ISR, mediated via JA/ET signaling pathways (Figure 3) [79].

### 3.5. Biotic and Abiotic Stress Mitigation Mechanisms by Trichoderma spp.

*Trichoderma* spp.-based bioactive compounds and elicitors are used as inoculants for phytostimulation. They interact with host plants through roots, enhancing tolerance to abiotic stresses such as drought and salinity [80]. *Trichoderma* spp. interacts with plant roots through recognition, attachment, penetration, colonization, and nutrient transfer [81]. *Trichoderma* spp.-based chlamydospores serve as active propagules in commercial formulations, such as SoilGard™, enhancing plant resistance to environmental stress [82]. A recent study reports that arbuscular mycorrhizal fungi (AMF) and *T. harzianum* (accession PV544806) influence maize responses to environmental stress. Maize plants were exposed to salinity (S_1_D_0_), drought (S_0_D_1_), or combined salinity–drought stress (S_1_D_1_), and treated with AMF alone (M_1_T_0_), *T. harzianum* alone (M_0_T_1_), or both microbes combined (M_1_T_1_). The results show that these microbial treatments activate specific stress-response pathways, highlighting the potential of AMF and *Trichoderma* spp. to enhance maize tolerance to climate change-related abiotic stresses [83]. Furthermore, *Trichoderma* combined with chitosan alleviates abiotic stress caused by heavy metals, including cadmium (Cd), improving pea seed germination under Cd toxicity. This combination also enhances physiological and biochemical parameters, including antioxidant enzyme activity and photosynthetic performance, supporting its suitability for cultivating plants in areas prone to Cd contamination [84]. Another study shows that *Trichoderma* inoculation reduces toxicity from Cd and Pb while promoting growth and physiological performance of *Vigna radiata* [85]. Application of certain *T. harzianum* strains to tomato seeds has been reported to accelerate germination and enhance tolerance to abiotic stresses, including drought, osmotic stress, salinity, chilling, and heat stress [62]. These strains have been reported to modulate the synthesis and signaling of key phytohormones, such as abscisic acid and indole-3-acetic acid (IAA), thereby enhancing plant physiological response to abiotic stresses [86].

### 3.6. Competition for Nutrients and Ecological Niches

Biological control in soil ecosystems is largely mediated by interactions between antagonistic and pathogenic fungi, involving mechanisms such as competition for nutrients and ecological niches, antibiosis, and mycoparasitism, particularly under nutrient-limited conditions. *Trichoderma* spp. can utilize a wide range of nitrogen-containing compounds, such as histamine, peptides, putrescine, and cytidine, enabling them to thrive under nutrient-limited conditions. In contrast, several phytopathogenic fungi, such as *Verticillium* spp., depend primarily on simple nitrogen sources, including urea, nitrite, and amino acid derivatives. This metabolic flexibility allows *Trichoderma* spp. to efficiently exploit available nutrients, thereby reducing resource availability for competing pathogens. In addition, *Trichoderma* spp. rapidly colonizes the rhizosphere and root surface, occupying ecological niches that prevent pathogens from establishing through competitive exclusion and improve their persistence and biocontrol efficacy in the soil ecosystem [87]. Certain *Trichoderma* spp. can produce siderophores, which are low molecular weight iron chelating compounds that sequester ferric iron (Fe^3+^) under iron-limited conditions. By reducing the availability of iron, an essential micronutrient for microbial growth and metabolism, siderophores restrict the proliferation of competing phytopathogens [88]. For example, *T. virens* XZ11-1 is capable of producing siderophores that inhibit the infection of banana fusarium wilt caused by *F. oxysporum* f. sp. *cubense* [89]. Another study reported that the synergistic interactions between *T. harzianum* NJAU4742 and soil microbiome enhance plant iron nutrition in calcareous soils [90].

### 3.7. Nutrient Solubilization and Growth Promotion by Trichoderma spp.

*Trichoderma* isolates secrete plant growth regulators and solubilize phosphates and micronutrients, including Fe, Mn, and Mg. They also secrete siderophores, exogenous enzymes, and vitamins supporting plant shoot growth. Certain *Trichoderma* strains exhibit “rhizosphere competence”, enabling them to colonize and proliferate in association with plant roots. However, their efficiency and persistence of rhizosphere colonization vary among species and strains and are influenced by soil characteristics and the host plant [12]. *Trichoderma* promotes root system development and plant nutrient acquisition through multiple mechanisms. The production of organic acids, specifically gluconic, citric, and fumaric acids, contributes to nutrient solubilization by lowering rhizosphere pH and enhancing the availability of insoluble mineral nutrients [91]. In addition, *Trichoderma* spp. facilitate nutrient mobilization through enzyme-mediated processes, microbial interactions, and competition with other microbes, thereby increasing nutrient cycling and availability. The improved uptake of macro- and micronutrients, together with enhanced root development and microbial-mediated defense responses, contribute to greater plant growth and stress tolerance [41]. Soil application of *T. harzianum* T447 has been reported to enhance the accumulation of essential nutrients, including Ca^2+^, Mg^2+^, P, Na^+^, and K^+^ in plant roots and shoots [92]. In addition to improving nutrient acquisition, *Trichoderma* spp. produce a range of bioactive metabolites, such as valerianol, 4-heptanone, and 3-octanone, which contribute to plant growth promotion [17]. Another study reported that several *Trichoderma* strains, including *T. atroviride* IMI206040, *T. virens* Gv29.8, and *T. asperellum* LU1370, have been shown to promote plant growth through the production of VOCs; however, LU1370 had a negative effect on plant growth promotion. This finding indicates that *Trichoderma*-mediated plant growth promotion is highly complex and variable, with its effects influenced by the fungal strain, experimental conditions, and environmental factors [93].

### 3.8. Phytohormonal Crosstalk in Trichoderma spp. Mediated Plant Defense

*Trichoderma* spp. modulate host plant phytohormonal networks, enhancing stress tolerance [94]. Plant–*Trichoderma* interactions induce production of phytoalexins, antimicrobial compounds that inhibit fungal growth [95]. Phytohormones are crucial for regulating complex and interconnected plant immune signaling pathways, facilitating rapid defense responses and adaptability to diverse environmental conditions. Yedidia et al. (1999) [15] reported that root colonization by *T. asperellum* induces systemic resistance in cucumber against Angular leaf spot caused by *Pseudomonas syringae* pv. *lachrymans*. The enhanced resistance was associated with increased expression of defense-related genes, including phenylalanine ammonia-lyase (PAL), a key enzyme in the phenylpropanoid pathway, and hydroxyperoxide lyase (HPL), an enzyme involved in the lipoxygenase pathway. Activation of these defense pathways promoted the biosynthesis and accumulation of phytoalexins, thereby enhancing the plant’s systemic defense against pathogen infection [96]. Phytoalexins are low-molecular-weight, inducible antimicrobial secondary metabolites that are rapidly synthesized at sites of infection in response to pathogen attack [97]. Another study demonstrated that two beneficial fungi, arbuscular mycorrhizal fungi and *Trichoderma*, modulate tomato plant metabolism by altering phytohormone levels. Specifically, treatment with these fungi increased the accumulation of auxins, cytokinins, and jasmonates, with the greatest increases observed in plants inoculated with *Trichoderma* spp. [98].

## 4. Formulation of *Trichoderma* spp.-Based Biocontrol Agents

**Figure 4:**
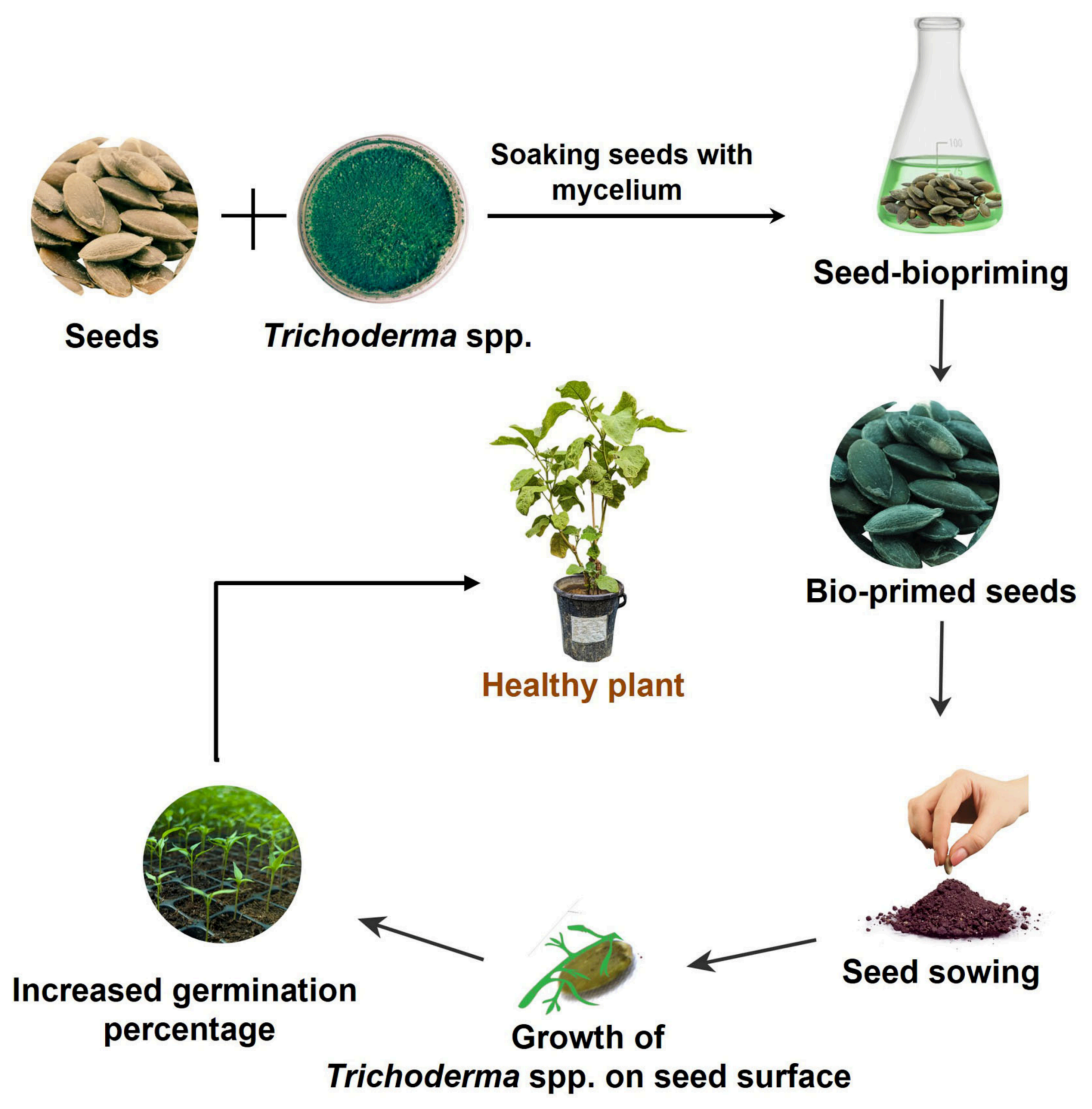
Schematic representation of seed biopriming with *Trichoderma* spp. Surface-sterilized seeds are soaked in a mycelial suspension to produce bio-primed seeds. The subsequent colonization of the seed surface by *Trichoderma* spp. enhances seed germination, promotes seedling establishment, and supports healthy plant growth.

**Figure 5:**
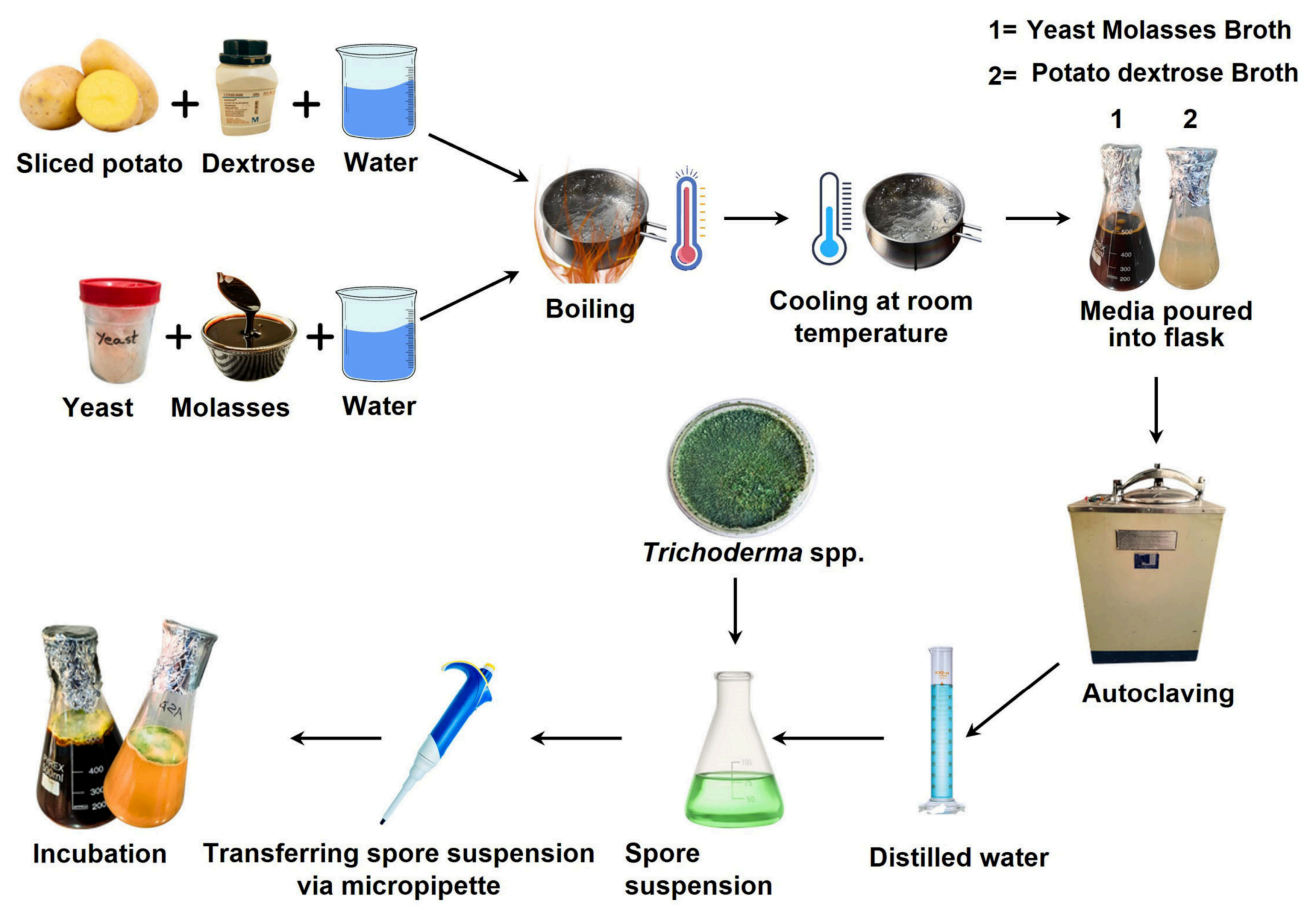
Preparation of enhanced *Trichoderma* spp.-based liquid formulations using yeast–molasses broth and potato dextrose broth.

*Trichoderma* spp. are cultivated on organic substrates and applied for crop disease management and formulated as pellets, dusts, powders, or emulsifiable liquids. Culture can be grown in liquid media, mixed with talc powder at a 1:2 ratio, and dried to 8% moisture under shade. These formulations have a shelf life of 3–4 months [99]. *Trichoderma* formulations, including powders, granules, emulsions, and suspensions, are widely used in various applications, such as dipping, spraying, root drenching, seed treatments, irrigation, and hydroponic systems [100]. Solid formations, such as pellets and dry flowables, are directly applied to the soil during sowing or transplanting [20]. However, successful large-scale application depends not only on biocontrol efficacy but also on efficient mass production and formulation stability (Figure 4 and Figure 5).

### 4.1. Solid-State Fermentation of Trichoderma spp.

Solid-state fermentation (SSF) is widely used for the large-scale production of *Trichoderma* spp. as it supports high spore yields using inexpensive solid. Frequently used substrates include sterilized compost, farmyard manure, rice bran, or cocopeat. The substrate should maintain optimal moisture to facilitate fungal growth while avoiding excess moisture that may create anaerobic conditions [101,102]. These formulations are applied directly to the soil to suppress soil-borne inoculum. The development of a stable formulation involves selecting an effective *Trichoderma* strain and an appropriate carrier substrate, followed by inoculation and incubation under controlled moisture, aeration, and temperature conditions until complete fungal colonization is achieved. The colonized substrate is subsequently dried to improve the storage stability and packaged in breathable containers to preserve spore viability before application to the soil for the management of soil-borne pathogens.

A semi-solid formulation of *T. harzianum* was prepared by combining 200 g of solid culture with 45 g citric acid, 45 g ascorbic acid, 100 g sodium benzoate, 100 g potassium sorbate, and 200 g xanthan gum. Citric and ascorbic acids reduced the formulation pH to 4.5–4.8, thereby limiting excessive CO_2_ production during fermentation, while xanthan gum stabilizes the culture and nutrient matrix [103]. A rice flour-based granular formulation of *T. asperellioides* CMAA1584 provides a cost-effective and scalable approach for biocontrol production. Rice flour, an inexpensive milling byproduct, supports high conidial yields (2.6 × 10^9^ colony-forming units (CFU)/g^−1^ in 14 days) when supplemented with 0.1% nitrogen, with hydrolyzed yeast proving most effective. The extruded granules function as micro-bioreactors, enabling in situ conidiation and enhancing secondary sporulation after rehydration. Formulations such as GControl and GBentonite exhibit marked ambient stability (>3 months), while GBentonite and GOrganic compost + Break-Thru remain viable for approximately 24 months under refrigeration. All formulations effectively suppress *S. sclerotiorum* (79–94% inhibition), even after prolonged storage, demonstrating improved shelf life and consistent biocontrol efficacy [104].

Agricultural digestate generated from anaerobic biogas production and enriched with food waste has been successfully utilized as a substrate for SSF of *T. reesei* RUT-C30 and *T. atroviride* Ta13, enabling the sustainable production of value-added bioproducts intended for biostimulant applications. Maximum fungal growth was achieved after 6 days for *T. reesei* and 3 days for *T. atroviride*. Both species produced cellulase, esterase and citric and malic acid, whereas *T. atroviride* additionally synthesized gibberellins and oxylipins, as confirmed by liquid chromatography-quadrupole time-of-flight mass spectrometry (UPLC-QTOF-MS). Furthermore, crude extracts obtained from *T. atroviride* SSF significantly enhanced tomato seed germination and root elongation, demonstrating their potential as plant biostimulants [105]. Another study reported that wheat bran-based formulations of *Trichoderma* spp. was developed and evaluated for the biological control of onion white rot caused by *Sclerotium* cepivorum. Among the five tested species (*T. album*, *T. harzianum*, *T. koningii*, *T. viride*, *T. virens*), *T. harzianum* and *T. koningii* exhibited the strongest antagonistic activity and provided the greatest reduction in disease severity under both greenhouse and field conditions. The improved disease resistance was positively associated with the increased activities of defense-related enzymes, such as peroxidase, polyphenol oxidase, and chitinase [106].

### 4.2. Liquid-Based Formulations of Trichoderma spp. for Agricultural Use

Liquid formulations are widely used in agricultural biocontrol applications by suspending or emulsifying *Trichoderma* spores or mycelia in water- or oil-based media [96]. Among various liquid media evaluated, potato dextrose broth (PDB) supports optimal growth of *Trichoderma* spp. Under greenhouse conditions, *T. harzianum* isolate T39 effectively controls cucumber foliar pathogens, including *B. cinerea*, *Pseudoperonospora cubensis*, *S. sclerotiorum*, and *S. fusca* [107]. In another study, laboratory media and locally available food grains were assessed as cost-effective substrates for the large-scale production of the local *T. viride* strain KBN-24. The results show that PDB and potato dextrose agar (PDA) produced the highest biomass yields of the *T. viride* strain KBN-24 [108]. Among the grain substrates, rice, wheat, and maize supported fungal growth, with rice producing the highest biomass. Mass multiplication of *T. viride* KBN-24 was evaluated using Sabouraud dextrose agar (SDA) and PDA. Biomass production was significantly higher on PDA (0.54 g plate ^−1^) than on SDA (0.37 g plate ^−1^). Among the laboratory media tested, PDA and PDB supported the highest biomass production of the *T. viride* KBN-24 strain. Similarly, among the grain substrates, rice was the most suitable for fungal growth, producing the highest biomass (148.04 g), followed by wheat (126.87 g), whereas maize resulted in the lowest biomass yield [109]. Furthermore, molasses yeast extract broth has been identified as the most effective medium for fermenter-based mass production of *T. viride*, with the maximum biomass achieved after 72 h of fermentation. Although liquid formulations are widely adopted owing to their ease of preparation and application, their long-term storage stability remains a major challenge. The shelf life of liquid formulations is influenced by several factors, including storage temperature, formulation additives (e.g., osmoprotectants and stabilizers), and packaging systems, which affect spore viability during storage. Under conventional storage conditions, liquid formulations generally exhibit a greater decline in viable propagules than solid carrier-based formulations. For example, after 180 days of storage, liquid formulations retained approximately 10^4^ CFU mL^−1^, whereas solid carrier systems maintained approximately 10^6^ CFU g^−1^, reflecting the superior protective environment provided by solid carriers [30].

### 4.3. Oil-Based Formulations

Oil-based formulations are based on mineral- or vegetable oils, represent an important class of biocontrol products in which microbial agents or bioactive compounds are suspended or dispersed in an oil phase. These formulations are commonly prepared as oil dispersions or emulsifiable concentrates [30,110]. Both vegetable- and mineral oil-based formulations have demonstrated excellent potential for delivering entomopathogenic fungi in biocontrol applications. Compared with aqueous suspensions, oil-based formulations generally provide greater thermal stability, enhance the survival of fungal propagules during storage, and improve resistance to environmental stresses, thereby contributing to better persistence and efficacy under field conditions [30]. Various carrier formulations, including canola oil, neem oil, paraffin oil, soybean oil, and glycerine, were used to deliver *T. harzianum* isolate spores [111]. For example, paraffin oil-based formulation of *T. viride* maintained high viability during storage, with the conidial population decreasing only from 2.8 × 10^9^ to 1.8 × 10^9^ CFU mL^−1^ after six months at 27 °C. The formulation also exhibited strong antagonistic activity, inhibiting the growth of *F. oxysporum* f. sp. *ciceri* and *Sclerotium rolfsii* by approximately 79–80% and *Rhizoctonia bataticola* by approximately 85%, demonstrating that oil-based formulations can effectively preserve microbial viability while maintaining biocontrol efficacy under storage conditions [112]. Oil-based emulsions formulated with commercial vegetable oils (Vatel^®^ and Surfatron^®)^, alongside the mineral oil Aceite Blanco^®^, containing *T. asperellum* TV190 conidia, markedly improve antagonistic efficacy against *R. solani* under greenhouse conditions compared to aqueous spore suspensions. Additionally, conidial survival following UV exposure is significantly greater in oil-based formulations (56–63% for vegetable oil emulsions) than in water-based controls (8–12%), thereby demonstrating the protective role of oil carriers against UV-induced damage [30,113]. The emulsions were prepared using a mixture of 20 mL Vatel^®^ vegetable oil, 10 mL Surfatron^®^, and 970 mL distilled water. For mineral oil formulations, Aceite Blanco^®^ was emulsified in water at a concentration of 20 mL L^−1^. A 1% (*w*/*v*) lignosulfonate solution was incorporated as a UV-protective additive. The spore concentration of *T. asperellum* TV190 was standardized to 1 × 10^6^ conidia mL^−1^ [113]. However, oil-based formulations also have limitations, including potential compatibility issues with certain microbial biocontrol agents, higher production costs, and the need for careful optimization of oil type and emulsifier composition to maintain microbial viability and long-term storage stability [114].

### 4.4. Vermiculite, Biochar, and Alginate-Based Formulations

Vermiculite is a phyllosilicate mineral belonging to the mica group, which includes minerals such as micas, chlorite, serpentine, talc, and clay [30]. Terralite^®^, a commercial horticultural-grade expanded vermiculite, is widely used as a solid carrier in fungal biocontrol formulations because of its high porosity, moisture-retention capacity, chemical inertness, and lightweight properties. In a seminal study, Lewis et al. (1991) used Terralite^®^ grade 4 vermiculite as the base carrier and combined it with wheat bran and conidia of several *Trichoderma* isolates to develop a stable solid formulation [115]. Early studies further demonstrate that vermiculite effectively protects *Trichoderma* conidia from desiccation and temperature stress, leading to improved shelf life and enhanced rhizosphere establishment compared to unformulated propagules. In these formulations, a concentrated *Trichoderma* spore suspension is mixed with vermiculite until the desired CFU concentration is achieved [116].

Biochar is a lightweight and extremely porous material produced from biomass pyrolysis. The final composition of ash and carbon varies depending on the type of biomass used and the production technique [117]. Biochar is often produced from feedstocks, such as corn residues, rice husks, fruit peels, agricultural waste wood, sludge, and microalgae [118].

Alginate is a non-toxic and biodegradable hydrocolloid capable of forming thermally stable hydrogel beads in the presence of divalent cations, including calcium. These materials are widely used to encapsulate medicines, oils, hormones, microbial cells, enzymes, herbal extracts, and other bioactive substances [119]. In submerged fermentation systems, *T. harzianum* propagules were successfully encapsulated in calcium alginate beads of varying sizes. Encapsulation of *Trichoderma* propagules in calcium alginate beads provides a highly protective microenvironment that enhances long-term viability and enables controlled release [30]. The beads retained a minimum viability value of 70% after 2 years of storage. The capsules with 1.5 ± 0.3 mm diameter exhibited the highest counts of free conidia, indicating the positive effect of Ca^2+^ ions on sporulation rates [120]. The presence of Ca^2+^ ions stabilizes the hydrogel matrix and positively influences sporulation rates and enzymatic activities associated with biocontrol mechanisms. Furthermore, alginate encapsulation enhances resistance to desiccation stress and UV radiation, making it a promising technology for developing next-generation sustainable bioformulations [30]. A *Trichoderma* suspension with a concentration of 20 × 10^10^ mL^−1^ was combined with a 0.6% sodium alginate solution at a ratio of 1:4. The mixture was then dripped into a beaker containing 1.5% CaCl_2_ and maintained at room temperature for 30 min to allow bead formation. The resulting CaCl_2_ beads, approximately 3 mm in diameter, were filtered and dried under sterile conditions. The alginate-coated *Trichoderma* beads were subsequently stored in plastic bottles containing sterile distilled water [121].

## 5. Shelf Life of Formulation Based on *Trichoderma* spp.

Under modified atmospheric conditions, different combinations of oxygen, carbon dioxide, and nitrogen may act as barriers against light, moisture, and oxidative damage. Additionally, vacuum packaging may extend the storage stability of *Trichoderma* [122]. The incorporation of stabilizing agents, such as glycerol in liquid formulations and optimization of carrier moisture content in dry formulations, is also a critical determinant of long-term propagule viability [30]. The choice of formulation for *Trichoderma* may significantly influence its shelf life. Additionally, appropriate storage conditions and packaging methods must be considered to optimize the preservation duration of *Trichoderma* formulations. The incorporation of glycerol (3–9%) into liquid formulations significantly extends the shelf life of *Trichoderma* spp. from 7 months to 10 months by reducing desiccation stress in the inoculant and maintaining viability levels above 2 × 10^6^ CFU g^−1^. In contrast, formulations prepared without glycerol exhibit a shorter shelf life of only 4–5 months. Furthermore, bioefficacy assays demonstrate that even after 12 months of storage, formulations produced with glycerol supplementation at 3% or 6% in the production medium effectively protect tomato plants against *Fusarium* wilt, thereby reducing disease incidence by 44–50% [123]. *T. viride* was produced in molasses-soy flour broth using liquid fermentation technology, with maximum biomass obtained after 72 h under optimal conditions. Antifoaming agents initially comprised polyethylene glycol; however, silicone was later adopted to reduce production costs. After harvesting, *T. viride* was formulated into de-oiled castor cake, gypsum, talc powder, vermicompost, and farmyard manure. These formulations maintain shelf life for 220 days, 190 days, and 180 days, respectively, supporting *T. viride* growth during storage. In contrast, talc powder- and gypsum-based formulations experience a decline in CFU/g^−1^ after 80 days of storage [124]. Vermiculite-based *Trichoderma* formulations retain viable propagule counts ranging from 10^6^ CFU g^−1^ to 10^8^ CFU g^−1^ for 6–12 months under ambient storage conditions, depending on moisture content, carrier composition, and storage temperature. Compared to liquid formulations, vermiculite-based dry formulations exhibit superior shelf-life, ease of handling, and suitability for soil and nursery applications. However, optimizing moisture levels and mitigating dust formation remain critical factors influencing the long-term viability and commercial scalability of these formulations. For example, a bentonite–vermiculite-based formulation of *T. harzianum* significantly enhances survival and biocontrol efficacy compared to a non-formulated conidial suspension when applied to melon plants under nursery conditions. The effectiveness of *T. harzianum* directly depends on the formulation type, as the carrier strongly influences its persistence in peat substrates. The formulated product maintained its initial inoculum viability (CFU count) for approximately 7–8 weeks, whereas non-formulated conidia exhibited a nearly 100-fold reduction in viable propagules. Consequently, plants treated with the bentonite–vermiculite formulation exhibit greater shoot biomass and enhanced resistance to *Fusarium* wilt in a seedling nursery of melon plants [125].

## 6. Challenges, Biosafety Concerns, and Limitations in the Application of *Trichoderma* spp.-Based Biocontrol Agents

A major challenge in producing *Trichoderma* spores in liquid media is maintaining high spore concentration and viability over time while preventing contamination and aggregation. Factors influencing spore production and quality include the composition of the liquid medium, pH, temperature, aeration, agitation, inoculum size, incubation time, and harvesting and storage methods. Different *Trichoderma* strains may require distinct optimal conditions for spore production, which should be adjusted based on the scale and intended purpose of production. Before the field application of *Trichoderma*-based products, these factors must be carefully considered to ensure maximal effectiveness [126].

Despite the extensive use of *Trichoderma* spp. as beneficial biocontrol agents and plant growth promoters, certain strains exhibit pathogenic potential and represent important exceptions that must be considered during their application. *Trichoderma* spp. are rarely associated with plant diseases. However, *T. hamatum* has been reported to cause postharvest bulb rot in *Lilium davidii* var. *willmottiae* in China [127]. Similarly, *T. lixii* was recently reported to cause leaf spot disease in *Ceropegia vincifolia* Hook in India [128]. *T. longibrachiatum* has been reported to cause leaf black circular spot disease on *Dendrobium nobile*. This plant is a medicinal orchid whose major bioactive alkaloid, dendrobine, has been investigated for its potential therapeutic effect in the treatment of cardiovascular and respiratory diseases [129]. In addition, *T. afroharzianum* has been reported as the causal agent of ear rot disease in maize (*Zea mays* L.) in Europe with significant yield loss [130].

In mushroom cultivation systems, some *Trichoderma* strains can cause green mold disease, leading to significant reductions in the yield of cultivated mushrooms, including champignon (button), oyster, and shiitake mushrooms [131]. These infections are primarily associated with *T. aggressivum* subspecies, which are highly adapted to mushroom production environments. Similarly, green mold disease in cultivated oyster mushrooms (*Pleurotus ostreatus*) has been linked mainly to *T. pleuroticola* and *T. pleuroti* [132]. These cases highlight the importance of strain-level identification and safety assessment, as beneficial and pathogenic traits within the genus *Trichoderma* are highly strain dependent. *Trichoderma*-associated green mold outbreaks cause substantial crop losses in various countries, including the UK, Netherlands, North America, France, Spain, Hungary, Poland, Croatia, Mexico, and Australia. For the first time, significant *Trichoderma* infections on maize ears were observed in a field in southern Germany. Samples of the infected maize cobs were collected, and the fungus was isolated in pure culture and initially identified through microscopic examination as *T. afroharzianum* [130].

Beyond plant-associated infections, certain *Trichoderma* spp. are implicated in human health complications, particularly respiratory tract infections. *Trichoderma* infections are often diagnosed at advanced stages and may be difficult to treat, making them potential opportunistic pathogens in immunocompromised hosts. *T. longibrachiatum*, capable of growth at 37 °C, is associated with clinical infections in humans [133].

Moreover, the effectiveness of *Trichoderma* spp.-based products is strongly influenced by environmental conditions, including climate, soil physiological properties, and the composition of native microbial communities. Temperature, soil moisture, pH, salinity, and nutrient availability can affect spore survival, rhizosphere colonization, metabolite production, and biocontrol efficacy. These limitations highlight the importance of selecting locally adapted strains, optimizing formulations, and conducting region-and crop-specific field evaluations before large-scale agricultural application [134].

## 7. Next-Generation Biofertilizers and Biocontrol Agents

Recent advances in omics technologies have significantly enhanced our understanding of plant growth-promoting microorganisms and accelerated the development of more effective microbial inoculants. Genomics, through whole-genome sequencing (WGS), genome annotation, and genome-editing tools, such as CRISPR-Cas9, enables the identification of genes associated with nitrogen fixation, nutrient acquisition, antimicrobial metabolite biosynthesis, abiotic stress tolerance, and plant growth promotion [135]. These insights facilitate the development of genetically improved microbial strains with enhanced biocontrol and plant growth-promoting capabilities. To date, genome sequencing efforts have been conducted for more than 200 *Trichoderma* spp. and strains, revealing extensive genomic diversity within the genus (https://www.ncbi.nlm.nih.gov/datasets/genome/?taxon=5543 (accessed on 20 July 2026).

Comparative genomic analyses have shown that this diversity is reflected in species-specific repertoires of genes involved in secondary metabolism, mycoparasitism, nutrient acquisition, and rhizosphere colonization. For example, *T. atroviride* contains numerous secondary metabolite biosynthetic gene clusters, which contribute to the production of diverse bioactive compounds. In contrast, *T. virens* possesses an expanded repertoire of gliotoxin biosynthetic gene clusters, enabling the production of gliotoxin, an antifungal secondary metabolite. Moreover, *T. virens* genome encodes genes for the biosynthesis of carbohydrate-active enzymes (CAZymes), which facilitate the degradation of plant cell wall polysaccharides, improve nutrient acquisition, and enhance rhizosphere colonization [136,137].

Metagenomics complements genomic studies by employing 16S rRNA gene sequencing, shotgun metagenomics, and bioinformatics platforms to characterize microbial community composition and functional potential within the rhizosphere, thereby enabling the discovery of novel beneficial microorganisms and improving our understanding of complex microbial interactions.

Transcriptomics, using RNA sequencing (RNA-seq), quantitative reverse transcription PCR (RT-qPCR), and microarray analysis, provides comprehensive information on gene expression profiles during plant–microbe and microbe–pathogen interactions, allowing the identification of regulatory pathways involved in stress adaptation, root colonization, induced systemic resistance, and antagonistic activity [138].

Proteomics further expands this understanding by profiling proteins expressed by both microorganisms and host plants through techniques such as two-dimensional gel electrophoresis (2D-GE), liquid chromatography–tandem mass spectrometry (LC-MS/MS), and matrix-assisted laser desorption/ionization time-of-flight mass spectrometry (MALDI-TOF MS). Specific functional proteins, such as ACC deaminase, antioxidant enzymes, such as superoxide dismutase, catalase, and peroxidases, have been identified as important molecular markers associated with plant growth promotion and enhanced tolerance to abiotic stresses [139].

Metabolomics complements genomic and proteomic analyses by characterizing the diverse metabolites produced by beneficial microorganisms and plants using analytical platforms, such as gas chromatography–mass spectrometry (GC-MS), liquid chromatography–mass spectrometry (LC-MS), nuclear magnetic resonance (NMR) spectroscopy, and Fourier-transform infrared (FTIR) spectroscopy. This approach facilitates the identification of antimicrobial compounds, phytohormones, volatile organic compounds, and other bioactive metabolites that promote plant growth and suppress pathogens [140].

Collectively, the integration of multi-omics approaches provides a comprehensive framework for elucidating the molecular basis of plant–microbe interactions and supports the rational development of next-generation microbial biofertilizers and biocontrol agents.

## 8. Field Applications of *Trichoderma* spp.

**Figure 6:**
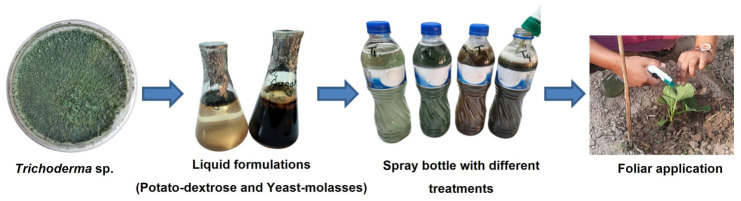
Foliar application of *Trichoderma* strains HSTUT-6 and HSTUT-8 formulated in potato–dextrose and yeast–molasses liquid media, followed by spraying the formulations onto plant foliage.

The application of *Trichoderma* spp. under field conditions has gained considerable attention as an environmentally friendly alternative to chemical pesticides and fertilizers. Although greenhouse and laboratory studies consistently demonstrate the biocontrol and plant growth-promoting potential of *Trichoderma*, field performance often varies because of fluctuations in environmental conditions, soil physicochemical properties, native microbial communities, crop genotype, and agricultural management practices. Consequently, recent research has focused on improving the consistency and reliability of *Trichoderma*-based bioinoculants under commercial farming conditions (Figure 6) [134,141].

Among the field studies reported to date, one notable example demonstrated that the application of *T. citrinoviride* Snef1910 provided more than 50% control efficacy against root-knot nematodes (*Meloidogyne incognita*) and significantly increased the biomass of tomato plants [142]. Another study reported that combining two *T. asperellum* strains CB-Pin-01 and NST-009 with CaCO_3_, a plant immune stimulant, reduced the incidence of tomato damping-off disease caused by *Pythium aphanidermatum* by 17.78% under greenhouse conditions [143]. The combined application of wettable powder formulations of *Trichoderma* strains with carbendazim (a broad-spectrum fungicide) provided significantly greater control of corynespora leaf spot disease (caused by *Corynespora cassiicola*) under field conditions, reducing disease incidence by 55–66%. In contrast, application of *Trichoderma* wettable powder alone showed limited disease control, while carbendazim alone reduced disease severity by only 40–45% [144]. *T. asperellum* 152–42 controls *Fusarium* root rot through integrated mechanisms involving direct antagonism, JA/ET-mediated immune priming, metabolic reprogramming, and rhizosphere microecological regulation, making it a promising biocontrol agent for sustainable alfalfa production [145].

## 9. Conclusions

*Trichoderma* spp. have emerged as multifunctional microorganisms that contribute to sustainable crop production through a combination of direct pathogen suppression, modulation of plant immune responses, and promotion of plant growth. Collectively, the evidence reviewed demonstrates that their biocontrol efficacy is governed by antibiosis, mycoparasitism, competition for nutrients and niches, ISR, and enhancement of plant resilience to abiotic and biotic stresses. Recent advances in genomics, transcriptomics, and metabolomics have substantially improved our understanding of the molecular basis of these interactions, revealing species and strain-specific differences in secondary metabolite biosynthesis, carbohydrate-active enzymes, signaling pathways, and rhizosphere competence that determine biocontrol preference.

Despite these advances, several challenges continue to limit the widespread and consistent application of *Trichoderma*-based bioinoculants. Variability in field performance resulting from environmental conditions, soil physicochemical properties, native microbial communities, and crop genotype remains a major constraint. In addition, differences among strains in colonizing ability, metabolite production, and, in rare cases, pathogenic potential underscore the importance of rigorous strain selection, biosafety assessment, and quality control during product development and commercialization.

Future research should focus on integrating multi-omics approaches with functional validation to identify superior strains and elucidate the regulatory networks underlying biocontrol and plant growth promotion. Further improvements in formulation technologies, delivery systems, and microbial consortia, together with long-term, region-specific field evaluations, will be essential to enhance the consistency, efficacy, and adoption of *Trichoderma*-based products. Such advances will facilitate the development of reliable biological alternatives to chemical pesticides and support the transition toward more resilient and sustainable agriculture systems.

## Figures and Tables

**Table 1:** Commercial formulations of *Trichoderma* spp. used for plant disease management.

Product Name	*Trichoderma* spp.	Primary Functions	Manufacturer	References
RootShield^®^	*T. harzianum* T-22; *T. virens* G-41	Controls pathogens including Phytophthora, Pythium, Fusarium, *Rhizoctonia*, *Cylindrocladium*, and *Thielaviopsis* spp. Enhances root hair development and improves water and nutrient uptake	BioWorks^®^, USA	[20,21,22]
PlantShield^®^	*T. harzianum* strain T-22 (Rifai; KRL-AG2)	Suppresses root pathogens, including *Pythium*, *Rhizoctonia*, *Fusarium*, and *Thielaviopsis* spp.	BioWorks^®^, USA	[20,22]
Eco-T^®^	*T. asperellum* KD	Protects against root diseases. Induces SAR in plants	Andermatt PHP (Pty) Ltd., South Africa	[20]
Binab^®^ TF-WP	*T. atroviride*; *T. polysporum*	Controls pathogens including *Fusarium*, *Pythium*, *Rhizoctonia*, and *Sclerotium* spp.	BINAB Bio-Innovation AB, Sweden	[20,23]
Promot^®^	*T. harzianum*; *T. koningii*	Produces enzymes degrading organic matter. Enhances soil biological activity and nutrient retention.	JH Biotech, Inc., USA	[20,23]
TRICHODERMA™ WP	*T. asperellum* isolate H22	Controls pathogens including *Fusarium*, *Rhizoctonia*, *Sclerotinia*, and *Pythium* spp.	Bioline Agrosciences Africa Ltd., Kenya	[20]
Tricho Plus^®^	*T. asperelloides* JM41R	Fungal inoculant for controlling root diseases and promoting plant growth	BASF South Africa (Pty) Ltd., South Africa	[20]
ASPERELLO^®^ T34	*T. asperellum* T34	Colonizes the root zone and forms a barrier against soil-borne fungal pathogens Promotes healthy root and plant growth	Biobest, Belgium	[24]
T-22™ HC	*T. harzianum* strain T-22	Protects against *Pythium*, *Rhizoctonia*, *Fusarium*, *Cylindrocladium*, and *Thielaviopsis* spp. Suitable for diverse crops, including cereals, cucurbits, herbs, and leafy vegetables.	BioWorks^®^; Koppert, The Netherlands	[25]
AkTRIvator^®^	*T. atroviride* IMI 206040; *T. afroharzianum* G.J.S. 08–137	Rapidly colonizes the root zone. Enhances root and root hair development, nutrient uptake, and resistance to pathogenic fungi.	CANNA, The Netherlands	[26]
Trichosan^®^	*T. koningii*	Suppresses soil-borne fungal pathogens, such as *Fusarium*, *Pythium*, and *Rhizoctonia* spp. Prevents damping-off, root rot, and wilt diseases.	Samarth Bio Tech Ltd., India	[27]
Vintec^®^	*T. atroviride* SC1	Protects against *B. cinerea* in crops, such as tomato.	Bi-PA, Belgium	[28]
Ecoderma	*T. viride*; *T. harzianum*	Protects against root rot and wilt diseases.	Morgo Biocontrol PvT. Ltd., India	[22]
Biofit	*T. viride*	Controls soil-borne pathogens, including *Pythium*, *Fusarium*, *Rhizoctonia*, *Sclerotium*, and other root-rot pathogens.	Ajay Biotech Ltd., India	[22]
SoilGard^®^ 12G	*T. virens* GL 20	Protects against root rot disease	Certis, USA	[22]
Trichogourd	*T. viride*	Protects against damping-off disease	Anu Biotech International Ltd., India	[22]
Bioderma	*T. harzianum*	Controls fungal pathogens, including *Fusarium*, *Phytophthora*, *Rhizoctonia*, and *Pythium* spp.	Ispahani Agro Limited, Bangladesh	[29]
Xedavir^®^	*T. asperellum* TV1	Antagonistic activity against root rot pathogens, including *Phthium* spp., *P. capsici*, *R. solani*, and *Verticillium* spp.	Xeda Italia S.r.I, Italy	[30]
Avengelus^®^	*T. atrobrunneum* T720	Reduces soil-borne and wood-associated fungal pathogens	MycoSolutions AG, Switzerland	[30]
Tenet™ WP	*T. asperellum* ICC012; *T. gamsii* ICC080	Controls soil-borne pathogens, including *Fusarium* and *Pythium* spp.	Isagro S.p.A., Italy	[30]

Abbreviations: SAR, systemic acquired resistance; WP, wettable powder; HC, high concentration formulation; TF-WP, wettable powder formulation containing multiple *Trichoderma* strains; IMI, International Mycological Institute strain designation; ICC, Isagro Culture Collection strain designation; SC, suspension concentrate (biopesticide formulation type); GL, strain designation used by the manufacturer.

**Table 2:** *Trichoderma* spp.-based biofertilizers and plant growth-promoting products.

Product Name	*Trichoderma* spp.	Primary Functions	Manufacturer (Company)	References
Biocure-F^®^	*T. viride*	Enhances root architecture	T. Stanes and Company Limited, Tamil Nadu, India	[20]
Trianum^®^-P	*T. harzianum* T-22	Suppresses pathogenic fungi and enhances nutrient uptake	Koppert B.V., Berkel en Rodenrijs, The Netherlands	[21,43]
Bio-Tricho	*T. harzianum*	Enhances nutrient availability and plant growth, particularly in acidic soils	Agro-Organics, New Delhi, India	[10,20,44]
Trichostar^®^	*T. harzianum* T58	Reduces the incidence and severity of root rot and other soil-borne diseases. Enhances nutrient uptake	Trentino Innovation, Italy	[21]
TrichoMax^®^	*T. asperellum* Ta.13	Produces IAA and promotes root growth	Biotor, San Isidro, Nicaragua	[20]
AgroGuard	*T. harzianum* DSM 14944	Promotes root growth and plant vigor	Live Systems Technology S.A., Colombia	[20,45]

Abbreviations: IAA, indole-3-acetic acid; DSM, Deutsche Sammlung von Mikroorganismen (German Collection of Microorganisms and Cell Cultures); B.V., Besloten Vennootschap (Dutch private limited liability company); S.A., Sociedad Anónima (corporation or public limited company).

**Table 3:** Effectiveness of *Trichoderma* spp. in managing target plant diseases.

Trichoderma spp.	Targeted Disease	Causal Agent	Crops	References
*T. harzianum*; *T. viride*	Damping-off	*Pythium* spp., *R. solani*, *Fusarium* spp.	Eggplant, okra, vegetables, chili, and cereals	[12,58,59]
*T. harzianum*; *T. asperellum*	Panama disease (*Fusarium* wilt)	*F. oxysporum* f. sp. *cubense*	Banana	[60]
*T. harzianum*; *T. virens*; *T. viride*	Collar rot	*Sclerotium rolfsii*	Lentil	[61]
*T. harzianum*	Gray mold	*B. cinerea*	Tomato	[60]
*T. hamatum* KUFA 0077	Sheath blight	*R. solani*	Rice	[62]
*T. longibrachiatum*; *T. virens*	White mold	*S. sclerotiorum*	Soybean, sunflower, and bean	[62]
*T. harzianum*; *T. lixii*; *T. afroharzianum*; *T. lentinulae*	Seedling blight	*Fusarium culmorum*	Wheat	[63]
*T. atroviride*; *T. asperellum*; *T. harzianum*	Root knot	*Meloidogyne incognita* *M. javanica*	Tomato	[60]
*T. viride*; *T. virens*; *T. harzianum*	Soft rot	*Pectobacterium carotovorum* subsp. *carotovorum*	Potato	[64]

## Data Availability

No datasets were generated or analyzed during the current study.
